# Vaccine adjuvant MF59 promotes the intranodal differentiation of antigen-loaded and activated monocyte-derived dendritic cells

**DOI:** 10.1371/journal.pone.0185843

**Published:** 2017-10-31

**Authors:** Rossella Cioncada, Marcella Maddaluno, Hoa Thi My Vo, Matthew Woodruff, Simona Tavarini, Chiara Sammicheli, Marco Tortoli, Alfredo Pezzicoli, Ennio De Gregorio, Michael C. Carroll, Ugo D’Oro, Diego Piccioli

**Affiliations:** 1 GSK Vaccines, Siena, Italy; 2 Boston Children’s Hospital, Harvard Medical School, Boston, MA, United States of America; Institut National de la Santeet de la Recherche Medicale (INSERM), FRANCE

## Abstract

MF59 is an oil-in-water emulsion adjuvant approved for human influenza vaccination in European Union. The mode of action of MF59 is not fully elucidated yet, but results from several years of investigation indicate that MF59 establishes an immunocompetent environment at injection site which promotes recruitment of immune cells, including antigen presenting cells (APCs), that are facilitated to engulf antigen and transport it to draining lymph node (dLN) where the antigen is accumulated. In vitro studies showed that MF59 promotes the differentiation of monocytes to dendritic cells (Mo-DCs). Since after immunization with MF59, monocytes are rapidly recruited both at the injection site and in dLN and appear to have a morphological change toward a DC-like phenotype, we asked whether MF59 could play a role in inducing differentiation of Mo-DC in vivo. To address this question we immunized mice with the auto-fluorescent protein Phycoerythrin (PE) as model antigen, in presence or absence of MF59. We measured the APC phenotype and their antigen uptake within dLNs, the antigen distribution within the dLN compartments and the humoral response to PE. In addition, using Ovalbumin as model antigen, we measured the capacity of dLN APCs to induce antigen-specific CD4 T cell proliferation. Here, we show, for the first time, that MF59 promotes differentiation of Mo-DCs within dLNs from intranodal recruited monocytes and we suggest that this differentiation could take place in the medullary compartment of the LN. In addition we show that the Mo-DC subset represents the major source of antigen-loaded and activated APCs within the dLN when immunizing with MF59. Interestingly, this finding correlates with the enhanced triggering of antigen-specific CD4 T cell response induced by LN APCs. This study therefore demonstrates that MF59 is able to promote an immunocompetent environment also directly within the dLN, offering a novel insight on the mechanism of action of vaccine adjuvants based on emulsions.

## Introduction

According to the statement of the World Health Organization reported in its web site, immunization via a vaccine, “is a proven tool for controlling and eliminating life-threatening infectious diseases and is estimated to avert between 2 and 3 million deaths each year. It is one of the most cost-effective health investments, with proven strategies that make it accessible to even the most hard-to-reach and vulnerable populations”. Vaccine adjuvants are substances co-administered with antigens to improve vaccine efficacy, especially in the case of vaccines made by inactivated pathogens or subunits of pathogens, which, in contrast to vaccines made with attenuated pathogens, can be poorly immunogenic[1–4]. After immunization, adjuvants may work as antigen delivery systems for immune cells and/or as immune-potentiators, stimulating the innate immune response which drives the magnitude and quality of the subsequent adaptive immune response[1–4]. Thus, vaccine adjuvants can have a key role to design the appropriate vaccine formulation in order to obtain an effective immunization[1–4]. Antigen presenting cells (APCs) and particularly dendritic cells (DCs) are critical immune cell types for eliciting an optimal antigen-specific immune response and exert their role being compartmentalized in specific areas of the lymph node (LN)[5–7]. DCs, that reside in the LN paracortex (called also T cell zone), can be considered strategic targets for immunization and consequently for the action of the adjuvants[5–9]. Therefore, adjuvants are very important for immunization strategies and consequently for the health of the mankind. Despite their importance, very few adjuvants are currently licensed for human vaccination[1–4]. MF59 is an oil-in-water emulsion adjuvant approved for human influenza vaccines[10], which, in preclinical studies, has been shown to have a multifunctional activity, because it is able to induce inflammation and immune cell recruitment at the injection site[11–14], to increase the number of antigen-loaded leukocytes within the draining LNs (dLNs)[13, 14], and to enhance antigen accumulation and retention within the dLNs, particularly in macrophage compartments (subcapsular sinus and medulla)[15]. In addition, MF59 enhances the transition from monocytes toward DCs (Mo-DCs), in vitro[16]. Mo-DCs have been used for a long time as a primary DC model to study the functionality of DCs and are considered a key DC subset for triggering and sustaining the T cell priming[17]. Although the phenotyping of the DC subsets is still evolving and the specific role of each DC subset in the induction of an immune response has not been completely clarified yet[8, 9, 18–20], Mo-DCs are believed to have a prominent role in the immune response following an inflammation process, such as that initiated by an immunization[17]. In fact, after vaccination, particularly in presence of an adjuvant, monocytes are recruited to the inflammation site and can differentiate into DCs, which mature, uptake the antigen and migrate into the dLNs where they can amplify the adaptive immune response, previously initiated by LN-resident DCs or by tissue-resident DCs which migrated earlier into the dLN[17]. In addition, Mo-DCs have been proposed as key target cells for vaccination strategies because they are able to drive follicular T helper cell response which is considered a critical step for the development of a long lasting immunity[21]. Although Mo-DCs remain the most important DC model for the human system, these cells cannot be unambiguously identified in the human blood[8, 9, 18, 20]. On the contrary, despite the complex phenotypic determination of DC population in the mouse tissues and lymphoid organs, murine Mo-DCs can be specifically identified in vivo using the CD64 (FcγRI) surface protein[22]. Since after immunization with MF59 monocytes are the APCs more rapidly recruited both at the injection site and in the dLN[13], we asked whether MF59 could play a role in promoting differentiation of Mo-DCs in vivo.

## Results

### MF59 promotes intranodal differentiation and transient accumulation of Mo-DCs

To detect Mo-DCs in vivo, we took advantage from the evidence that CD64 has been described as an unambiguous marker to identify these cells in the mouse immune system[20, 22].

We immunized mice in both legs with fluorescent protein Phycoerythrin (PE) as model antigen, in presence or absence of MF59 and using PBS as negative control. The fluorescence of the antigen made it possible to track the antigen-loaded cells[15]. Treated mice were sacrificed 15 minutes (15 min), 8 hours (8 hrs), 18 hours (18 hrs) and 3 days (3 d) after primary immunization and the dLNs were collected. LNs from the right legs were pooled and analyzed by flow cytometry, whereas LNs from the left legs were analyzed individually by confocal microscopy. Imaging studies of dLNs were performed because we wondered if MF59 may have an impact on the antigen distribution within the dLNs, on the organization of LN compartments and what was the localization of this adjuvant among the different zones of the LN[5–7].

**Fig 1:**
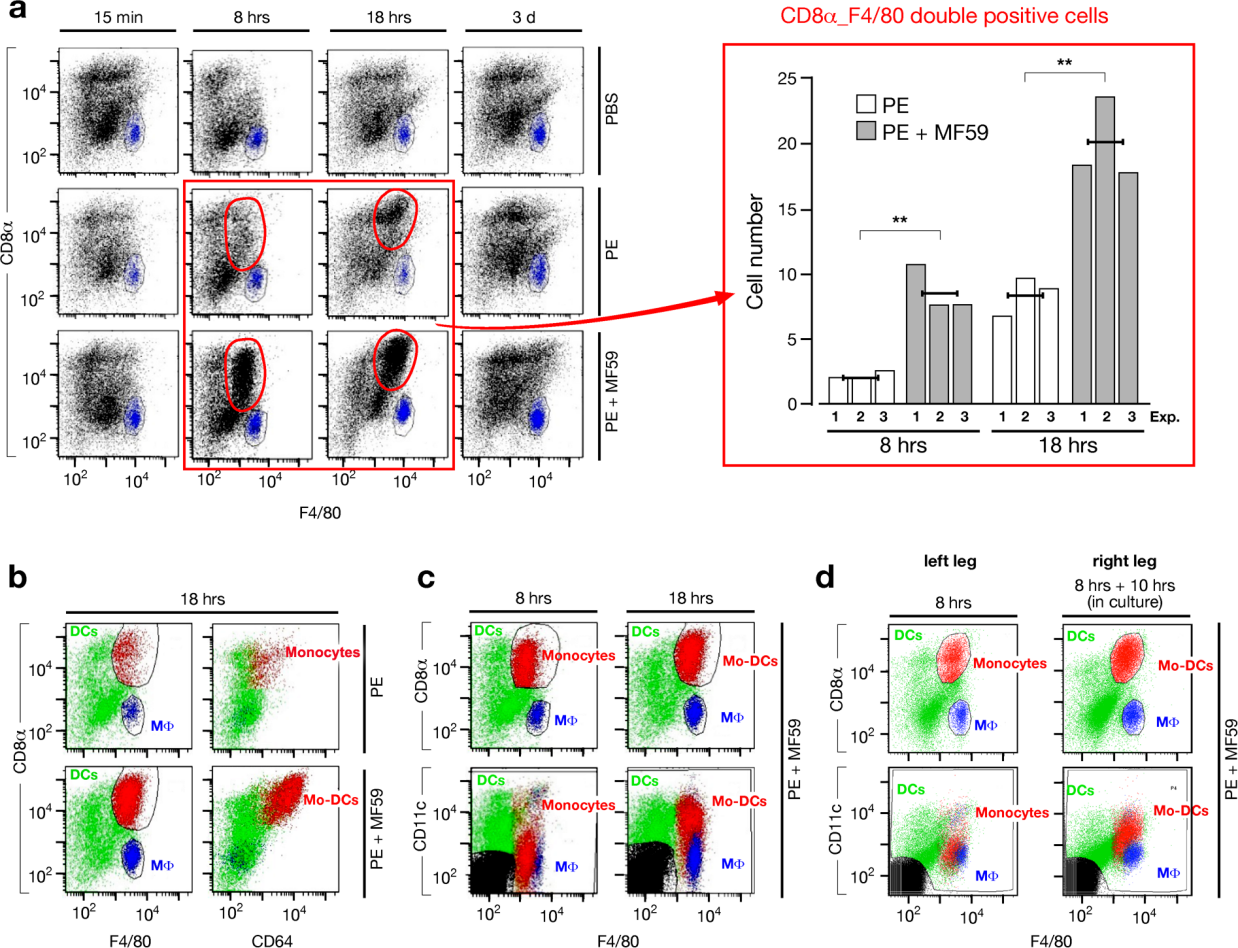
MF59 promotes differentiation and transient accumulation of Mo-DCs within the dLN. (**a**) Left panel: flow cytometry dot plots of dLN cell suspensions to identify APCs are reported for each time point and each treatment (as indicated). Macrophages (MΦ) are depicted in blue, whereas the rest of the APCs are in black. The transient appearance of a CD8α_F4/80 double positive APC subset (red gates) between 8 hrs and 18 hrs after immunization (red quadrant) is shown. Representative results of one experiment out of three are shown. Right panel: bar graph histogram that reports the number x10^3^ (per million of live/singlet cells acquired by flow cytometry) of CD8α_F4/80 double positive APCs at 8 hrs and 18 hrs after immunization with PE (white bars) and PE + MF59 (grey bars). Data from three independent experiments (single numbered bars) and the arithmetic mean of these values (black horizontal line) are shown. Statistical analysis: parametric one-tailed T-test was used to calculate the P-value. **P<0.01. (**b**) Flow cytometry dot plots of dLN cell suspensions obtained 18 hrs after immunization with PE or PE + MF59 are shown to analyze the expression of the surface protein CD64 on the different APC subsets. CD8α_F4/80 double positive APCs are depicted red, LN DCs in green and MΦs in blue. Representative results of one experiment out of the three independent experiments reported in panel (a) are shown. (**c**) Flow cytometry dot plots of dLN cell suspensions obtained 8 hrs and 18 hrs after immunization with MF59 reveal that the CD8α_F4/80 double positive APC subset (red cells) expresses the CD11c DC marker 18 hrs but not 8 hrs after the immunization. Representative results of one experiment out of the three reported in panel (a) are shown. (**d**) Flow cytometry dot plots of the dLN cell suspensions to identify APCs derived from mice immunized with MF59, whose LNs were explanted from both legs 8 hrs after the immunization, but: the LNs from the left legs (left dot plots) were immediately processed (8 hrs), whereas the LNs from the right legs (right dot plots) were processed after 10 hrs of in vitro culture (8 hrs + 10 hrs (in culture)). Representative results of one experiment out of three are shown.

The APCs were phenotypically characterized according to the reported gating strategy (S1 Fig), which is based on the current knowledge[8, 18, 19, 23, 24]. We observed a transient accumulation of a CD8α_F4/80 double positive APC subset between 8 and 18 hrs after immunization with MF59, revealed by the significant increase in the cell number, compared to mice immunized with PE alone, at the two time points (8 and 18 hrs after treatment) taken in consideration (Fig 1A). This cell accumulation is also observed injecting MF59 alone without any co-administered antigen. The accumulated CD8α_F4/80 double positive APC population (red cells) specifically expresses the Mo-DC marker CD64, compared to macrophages (MΦs; blue cells) and LN DCs (green cells) that are CD64 negative (Fig 1B), but, very interestingly, it displays the DC phenotype, via the CD11c marker expression, only 18 hrs after immunization with MF59 (Fig 1B and 1C, S2 and S3A Figs), whereas, at 8 hrs, both in presence or absence of MF59, this APC subset has a monocyte phenotype[8, 18, 19, 23, 24], being CD11c low or negative (Fig 1C, S2A and S3A Figs) and considering also the high expression of the Ly6-C monocyte marker (S3B Fig). Thus, the CD8α_F4/80 double positive APCs are monocytes 8 hrs and Mo-DCs 18 hrs after the immunization with MF59, whereas in absence of MF59 this cell population is always represented by monocytes. Consistently, CD64 is up-regulated 18 hrs after immunization with MF59, revealing a more mature Mo-DC phenotype (S3C Fig), and Mo-DCs display a high Ly6-C expression according to their monocyte origin (S3B Fig). Mo-DCs are also the APCs that exhibit a high and homogeneous expression of MHC class II molecules (S4 Fig). Summarizing, when immunizing with MF59, our phenotypic analysis discriminated the following four APC subsets: DCs (green) as CD11c^high^_F4/80^-/low^_CD8α^-to+^_CD64^-^_Ly6-C^+^; MΦs (blue) as CD11c^low^_F4/80^+^_CD8α^-^_CD64^-^_Ly6-C^-^; Monocytes (red) as CD11c^**-/low**^_F4/80^+^_CD8α^+^_CD64^**+**^_Ly6-C^high^ (8 hrs after immunization); Mo-DCs (red) as CD11c^**high**^_F4/80^+^_CD8α^+^_CD64^**+**^_Ly6-C^high^ (18 hrs after immunization). We thus concluded that MF59 promotes accumulation within the dLN of monocytes and Mo-DCs respectively 8 and 18 hrs after primary immunization, suggesting that MF59 induces intranodal differentiation of Mo-DCs. To support this hypothesis we performed an ex-vivo experiment in which we isolated the dLN 8 hrs after immunization and followed Mo-DC differentiation after an in vitro culture of the whole LN. Mice were immunized in both legs with PE or PE adjuvanted with MF59. Animals were sacrificed after 8 hrs and the popliteal dLNs from both legs were collected. The LNs from the left legs were immediately processed and analyzed by flow cytometry as described in the previous experiment, whereas LNs from the right legs were cultured intact and untouched for an additional 10 hrs and then analyzed by flow cytometry (Fig 1D). As expected and already described (Fig 1B), we found accumulation of monocytes in the dLNs analyzed 8 hrs after immunization with MF59 (left leg LNs) (Fig 1D, left dot plots). Interestingly, in the dLNs of the same animals explanted at the same time point (8 hrs) after immunization and cultured for 10 hrs (right leg LNs), we found accumulation of Mo-DCs similarly to what observed in the dLNs explanted and analyzed 18 hrs after immunization (Fig 1D, right dot plots and S3D Fig). This result unequivocally demonstrates that monocytes recruited at 8 hrs within the dLN of mice immunized with MF59, became DCs 10 hrs later, indicating that MF59 promotes intranodal differentiation of Mo-DCs.

The site of administration does not critically affect this phenomenon (S5A Fig), whereas the use of ALUM (another clinically approved adjuvant) induces Mo-DC accumulation only 3 d after the immunization (S5B and S5C Fig), indicating that this phenomenon is delayed when immunizing with ALUM.

### Mo-DCs are the major intranodal source of antigen-loaded and activated APCs, when immunizing with MF59

**Fig 2:**
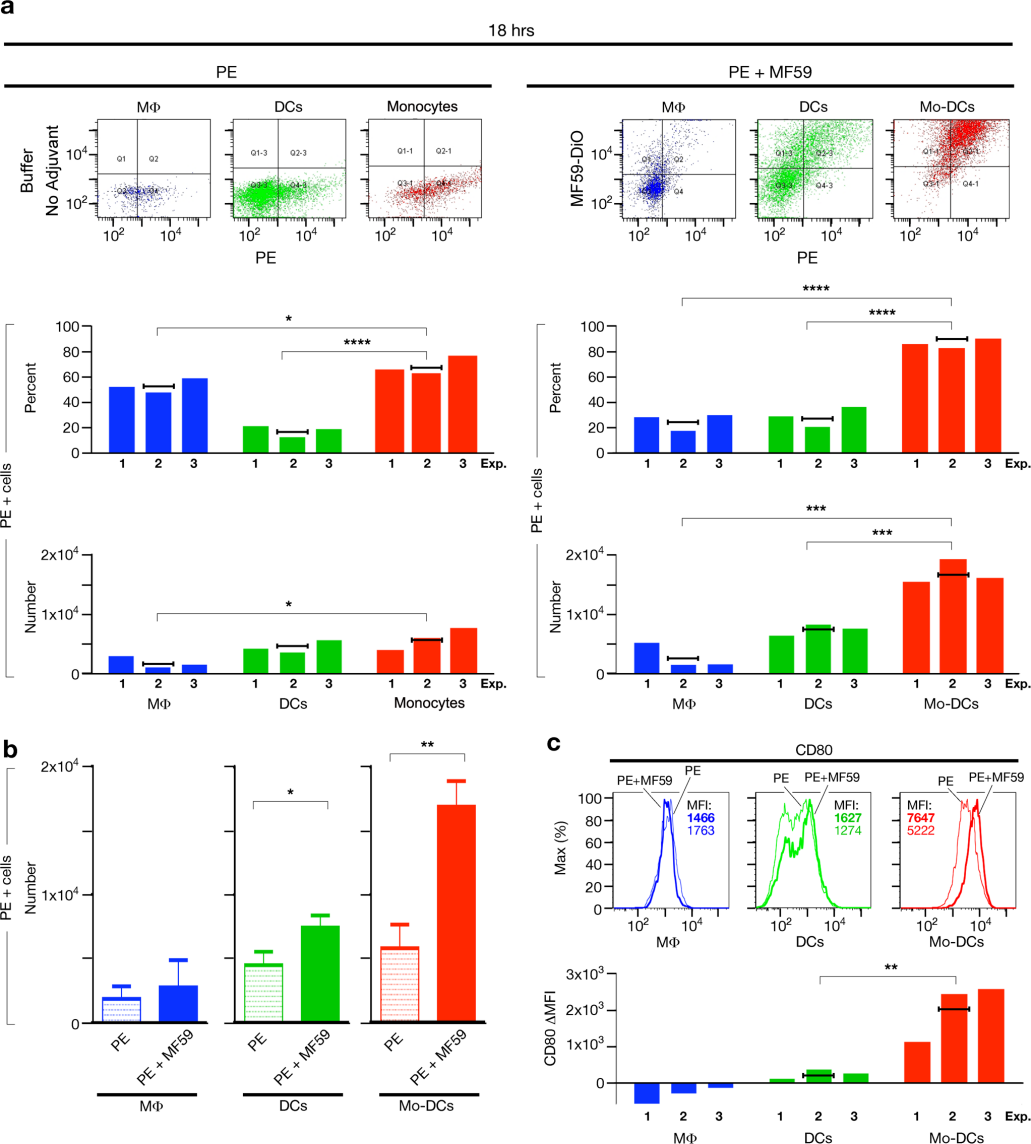
Mo-DCs are the major source of antigen-loaded and activated APCs within the dLN when immunizing with MF59. Popliteal dLN cell suspensions from mice immunized with PE or PE + MF59 analyzed by flow cytometry 18 hrs after immunization. (**a**) Upper panels: fow cytometry dot plots that show the uptake of PE and MF59 by MΦs (blue cells), DCs (green cells) and monocytes or Mo-DCs (red cells) from one representative experiment in mice immunized with PE or PE + fluorescently labelled-MF59 are reported as example. Middle and lower panels: bar graph histograms report the percentage (middle panels) and the number (per million of live/singlet cells acquired by flow cytometry) (lower panels) of PE positive cells in MΦs (blue bars), DCs (green bars) and monocytes or Mo-DCs (red bars). Data from three independent experiments (single numbered bars) and the arithmetic mean of these values (black horizontal line) are shown. Statistical analysis: parametric one–way ANOVA test (Dunnett’s multiple comparison using Mo-DCs as control column) has been applied to calculate the P-value.*P˂0.05; ***P˂0.001; ****P˂ 0.0001. (**b**) Bar graph histogram reports the average (+ standard deviation) number (per million of live/singlet cells acquired by flow cytometry) of PE positive MΦs (blue bars), DCs (green bars) and Mo-DCs vs. monocytes (red bars) in mice immunized with PE (dotted bars) or PE + MF59 (filled bars) in the three independent experiments reported in panel (a). Statistical analysis: parametric one-tailed T-test was used to calculate the P-value. *P˂0.05; **P<0.01. (**c**) Upper panels: flow cytometry histograms of CD80 expression by MΦs (blue), DCs (green) and Mo-DCs vs. monocytes (red) in mice immunized with PE (thin lines) or PE + MF59 (thick lines) from one experiment out of the three shown in panel (a). Lower panel: bar graph histogram shows the difference in CD80 Mean Fluorescence Intensity (MFI) of MΦs (blue bars), DCs (green bars) and Mo-DCs vs. monocytes (red bars) from mice PE immunized with and without MF59 (ΔMFI). Data of the same three independent experiments reported in panel (a) (single numbered bars) and the arithmetic mean of these values (black horizontal line), are shown. Statistical analysis: parametric one-tailed T-test was used to calculate the P-value. **P˂0.01.

Taking advantage of the intrinsic fluorescence of PE, we evaluated antigen uptake by APCs by measuring the percentage and the number of antigen-loaded cells (S6 Fig). First of all, 18 hrs after immunization, the majority of antigen-positive LN cells is represented by APCs, both in presence or absence of MF59 (S6A Fig). Secondly, the percentage and the number of antigen-loaded APCs are significantly enhanced following immunization with MF59 only at 18 hrs after treatment, when accumulation of Mo-DCs is observed (S6B Fig and Fig 1). An increase in number of antigen-loaded APCs in MF59-treated animals 8 hrs after immunization (in presence of monocyte accumulation) is observed, but it is not statistically significant due to the high variability (S6B Fig). Thirdly, when immunizing both in presence or absence of MF59, the percentage and the number of antigen-loaded APCs are generally higher at early time points, peak at 8 hrs after immunization (S6B Fig), and then decrease afterwards, but this decrease is slower in presence of MF59. We therefore concluded that MF59 induces a persistence of antigen positive APCs during the first 18 hrs, which correlates with a concomitant accumulation of Mo-DCs (S6 Fig and Fig 1). Consequently, we investigated what was the contribution of Mo-DCs in the enhancement of antigen-loaded APCs at 18 hrs following immunization. We found that, both monocytes and Mo-DCs, as entire cell populations, display a superior ability to engulf the antigen among APCs, because the percentage of antigen-positive cells in these populations is significantly higher compared to DCs and MΦs (Fig 2A, flow cytometry dot plots and upper bar graphs). Actually, MΦs display a reduced antigen engulfment in presence of MF59 (Fig 2A, flow cytometry dot plots and upper bar graph). Monocytes and Mo-DCs are cell populations with a similar ability to engulf antigen, although the trend of the antigen uptake is slightly higher in Mo-DCs than in monocytes (Fig 2A, upper bar graphs, red bars). At the same time, when immunizing with MF59, the Mo-DC population contains a significantly higher number of antigen-loaded cells compared to both DCs and MΦs (Fig 2A, lower right bar graph), whereas in absence of MF59, the number of antigen-loaded monocytes is superior only compared to MΦs and at lesser extent (Fig 2A, lower left bar graph). In addition, formulation with MF59 mainly enhances the number of antigen-loaded Mo-DCs compared to monocytes, because the increment in the number of antigen-loaded cells is higher in Mo-DCs versus monocytes than in DCs, when immunizing with MF59, whereas MΦs are not affected (Fig 2B). We thus concluded that, 18 hrs after immunization with MF59, Mo-DCs represent the most abundant antigen-loaded cell population within the dLN (Fig 2A and 2B and S6 Fig). In addition, using fluorescently labeled MF59 (which does not display an affected adjuvant capacity) together with PE antigen, we measured the percentage of antigen and MF59 positive cells and we found that, although the adjuvant emulsion is engulfed together with the antigen by all three APC subsets, the Mo-DC population is mostly double loaded with both antigen and adjuvant (Fig 2A, flow cytometry dot plots and S7 Fig) and contains the higher percentage of cells loaded with MF59. After immunization with ALUM, Mo-DCs appear roughly two days later compared to MF59 (S5C Fig), but at this time point they are barely loaded with the antigen (S5D Fig). Thus we suggest that these cells might not be able to contribute to the induction of an adaptive immune response. Since activated APCs with up-regulated co-stimulatory molecules are required for an optimal T cell response[17], we finally checked cell activation by measuring the expression of the co-stimulatory molecule CD80 on APCs within the dLN (Fig 2C). We found that Mo-DCs display the highest CD80 expression among the three APC subsets and a significantly increased CD80 up-regulation (based on CD80 expression of monocytes) after immunization with MF59 as compared to DCs and MΦs (that do not up-regulate CD80) (Fig 2C). Consistently with this observation, the Mo-DC population is the only APC subset which up-regulate also the co-stimulatory molecule CD86 (S8 Fig). All together these results demonstrate that, during the first 18 hrs after an immunization, the presence of MF59 adjuvant promotes accumulation and differentiation within the dLNs of Mo-DCs that, among APCs, display a more activated phenotype, a higher ability to engulf the antigen as a unique cell population and consequently increase the persistence of antigen-bearing APCs within the dLNs. Therefore, when immunizing with MF59, the Mo-DC population is the major source of antigen-loaded and activated APCs within the dLN.

### Antigen-loaded Mo-DCs could be localized in the medullary region of the dLN

**Fig 3:**
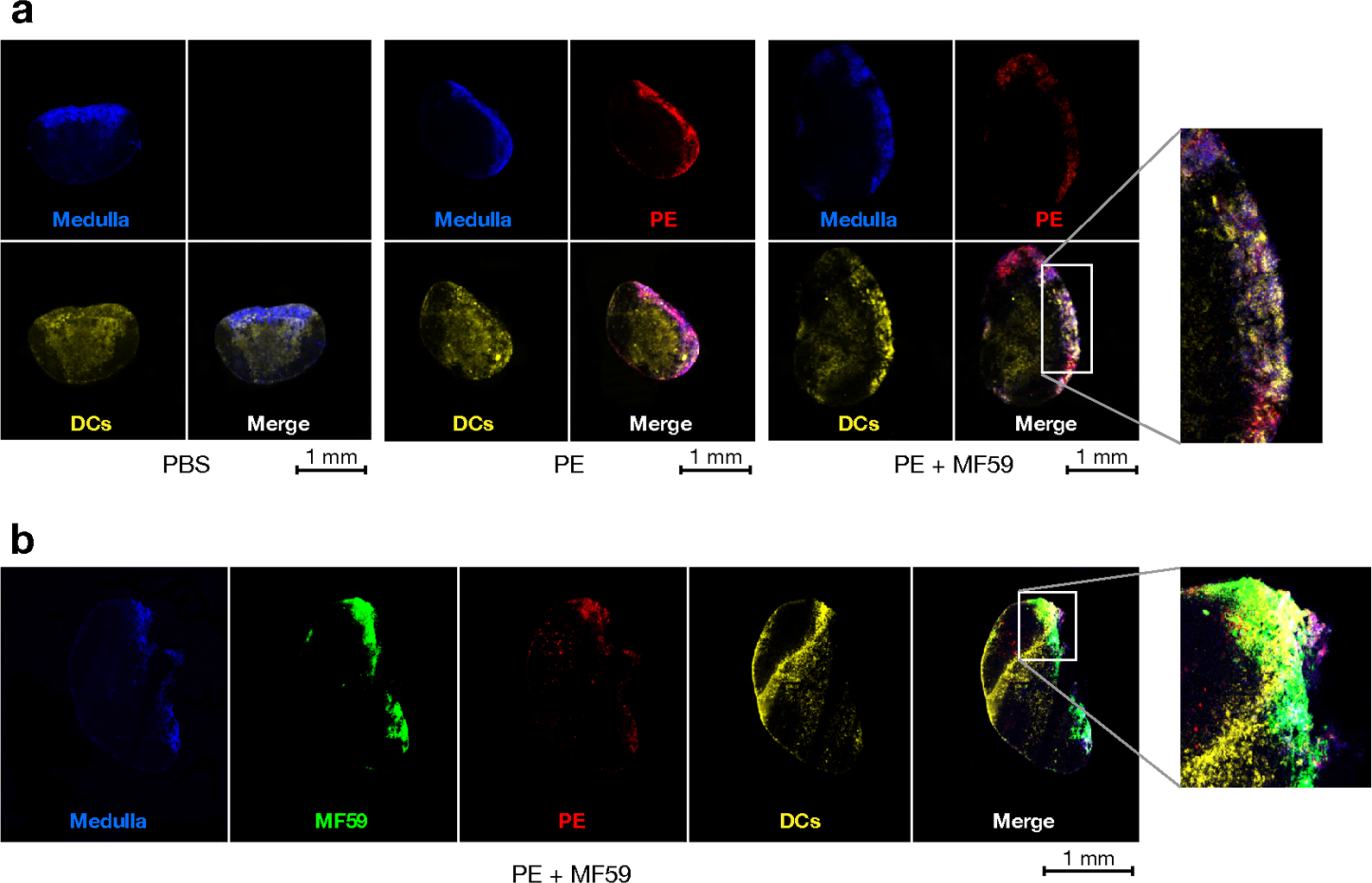
Co-localization of antigen, MF59 and DCs within the medullary compartment of the dLN. (**a**) Confocal microscopy images of dLNs collected from mice 18 hrs after treatment with PBS, PE or PE + MF59 and labeled to detect DCs and the medullary compartment of the LN. Signals which detect the LN medulla (blue, F4/80), PE (red) and DCs (yellow, CD11c) are shown separately and merged (as indicated). The magnification shows the co-localization of antigen and DCs within the LN medulla. The image of one section is shown, in each panel, as example of consecutive sections of a whole LN, which is representative of the organs of all immunized mice. Original magnification: 5X. Scale bar: 1 mm. Results of one representative experiment out of three are reported. (**b**) Confocal microscopy images of dLNs collected from mice 18 hrs after treatment with PE + fluorescently labelled-MF59 and labeled to detect DCs and the medullary compartment of the LN. Signals which detect the LN medulla (blue), MF59 (green), PE (red) and DCs (yellow) are shown separately and merged (as indicated). The magnification shows the co-localization of antigen, MF59 and DCs within the LN medulla. The image of one section is shown, in each panel, as example of consecutive sections of a whole LN, which is representative of the organs of all immunized mice. Original magnification: 5X. Scale bar: 1 mm. Representative results of one representative experiment out of two are reported.

Consistently with flow cytometry data, by confocal microscopy we detected antigen and MF59 in the dLN already 15 min after the injection, distributed along the subcapsular sinus and within the medullary area. We also used two-photon microscopy to provide, for the first time, a real time kinetic analysis of the arrival of an MF59-adjuvanted antigen in the dLN (S1 and S2 Videos). We found that both antigen and MF59 are rapidly (in less than a minute) translocated to the dLN (S1 and S2 Videos) and in this early phase they appear to transfer independently, confirming a previous observation[25]. By confocal microscopy, eight hours after immunization, both antigen and MF59 are prevalently detected within the medullary area. However, focusing our attention on the 18-hour time point after immunization, we observed that the antigen is still localized within the medullary compartment of the LN either in absence or in presence of MF59 (Fig 3A). Nevertheless, through CD11c labeling, after immunization with MF59 we observed a remarkable accumulation of DCs within the medullary area of the LN (Fig 3A). Furthermore, using labeled MF59, we observed that also the adjuvant is localized within the medullary compartment together with the antigen and DCs (Fig 3B). The co-localization of antigen and adjuvant within the medullary region was confirmed also by 3D reconstruction of the dLN analyzed by two-photon microscopy (S3 and S4 Videos). Thus, we demonstrated that, 18 hrs after immunization with MF59, both antigen and adjuvant accumulate within the medullary compartment of the dLN together with DCs.

Collectively, flow cytometry and confocal microscopy results of this study suggest that, when immunizing with MF59, the observed differentiation and accumulation of antigen/adjuvant loaded and activated Mo-DCs could take place within the medullary compartment of the dLN.

### APCs derived from dLNs of mice immunized with MF59 have an enhanced capacity to trigger antigen-specific CD4 T cell response

In order to correlate our finding with the adjuvant function of MF59, we analyzed the ability of APCs from dLNs of immunized mice to trigger antigen-specific CD4 T cell response[17]. We immunized mice with Ovalbumin (OVA) in presence or absence of MF59 and then cultured dLN APCs with CFSE-loaded OVA-specific CD4 T cells, measuring T cell proliferation by flow cytometry (S9 Fig). We discovered that the percentage of CFSE halving of OVA-specific CD4 T cells is significantly enhanced when the cells are cultured with dLN APCs obtained from mice immunized with MF59 adjuvant, revealing a potentiated triggering of antigen-specific CD4 T cell response. This result is consistent with the enhancement effect of MF59 on antibody response to OVA which has been already described[26]. In addition, we also confirmed that MF59 display adjuvant activity with our model antigen because it induces a significant increment of the humoral response to PE after immunization (S10 Fig) (either b.t. or i.m).

We can conclude that accumulation and differentiation of antigen-loaded and activated Mo-DCs within the dLN induced by MF59 correlate with the ability of dLN APCs to induce an enhanced antigen-specific T cell activation and, ultimately, with the adjuvant activity of MF59.

## Discussion

This study elucidates a new aspect of the mode of action of MF59 emulsion adjuvant, which until now was believed to promote the establishment of a transient “immunocompetent” environment at the injection site, resulting in the recruitment of APCs, which then take up antigen and adjuvant and transport them to dLNs, where they trigger the immune response[11–14, 16, 25, 27]. Although this proposed mechanism of action for MF59 is still valid, our study demonstrates that at least an additional mechanism of MF59 adjuvant activity exists, by which MF59 promotes an “immunocompetent” environment that induces differentiation of antigen-loaded and activated Mo-DCs, directly in the dLN.

A previous study demonstrated that monocytes are the first non-dispensable APCs for immune response to reach both injection site and dLN[13]. In fact, also neutrophils, that are more rapidly recruited into the injection site and dLN, are vehicles of antigen, but these cells have been demonstrated to be dispensable for the adjuvant activity of MF59[13]. The number of antigen/adjuvant double positive monocytes peaks at 17 hours from the treatment, at the injection site, whereas it peaks at 7 hours within the dLN, consistently with our findings[13]. In addition, only after immunization with MF59 these monocytes display a critical change in morphology toward a DC-like phenotype[14]. Based on these published observations, our current results, and the well described mechanism of trafficking of cells and substances within the dLN[5–7], we suggest the following non-exclusive hypothesis for the mode of action of MF59: after immunization with MF59, the adjuvant rapidly reaches the dLN where it stimulates local cells to produce soluble factors that induce monocyte recruitment and drive the differentiation of Mo-DCs; thus monocytes migrate from the circulation directly into the medulla of the LN where they engulf antigen and adjuvant and differentiate into DCs. Ultimately, we propose that the vaccine adjuvant MF59 promotes a stimulatory environment within the medullary compartment of the dLN which drives the differentiation of activated and antigen/adjuvant-loaded Mo-DCs.

In addition to this finding, we observed that dLN APCs from mice immunized with MF59 have an enhanced capacity to trigger antigen-specific CD4 T cell response. In this respect, it is noteworthy the fact that it has been recently discovered that Mo-DCs drive the follicular helper T cell differentiation, which is a critical event for developing a long lasting immunity[21]. Considering these last two findings, it is tempting to speculate that Mo-DC may play an essential role in triggering an adaptive immune response associated with an effective immunization and that intranodal differentiation of antigen-loaded and activated Mo-DCs may be one of the major mechanism driving MF59 adjuvant function, but further investigations are required to support this hypothesis. For example, CCR2 plays a key role in the monocyte egress from bone marrow into the blood circulation[22, 23]. In agreement with this, it has been found that, after immunization with MF59, the recruitment of mononuclear cells into the inflamed tissues is dramatically reduced in CCR2 KO mice[11]. Thus it would be important to evaluate if in these mice a significant reduction of Mo-DCs within the dLNs can be observed when immunizing with MF59 and if this observation is associated with a reduction in MF59 adjuvant capacity. These results would ultimately demonstrate that the intranodal differentiation of Mo-DCs is essential for MF59 to carry out its adjuvant function.

We think that if in vivo differentiated Mo-DCs represent the APC subpopulation which is necessary and sufficient to drive MF59 adjuvanticity, the elucidation of the molecular mechanisms triggering this differentiation could help to improve the efficacy of emulsion adjuvants and to identify new more effective adjuvants using Mo-DCs as target cells. For example, it has been described that MF59 increases the ATP release at injection site which modulates its adjuvant function[26]. It would be intriguing to investigate if MF59 induces the ATP release also within the dLN and if this release is involved at some stage of the Mo-DC differentiation and/or activation. Since the adjuvant capacity of MF59 is partially dependent on the adaptor molecule MyD88, but MF59 is not a TLR agonist[28], it would be interesting to examine if MyD88 is critical for a signal transduction pathway downstream to a receptor that works as a sensor for ATP or other intermediates of the ATP metabolism.

Whether antigen-loaded Mo-DCs play a role in the mode of action of other adjuvants, such as ALUM, has not been described. However, also ALUM adjuvant seems to promote the transient appearance of Mo-DCs within the dLN, but this occurs 3 days after the immunization, delayed compared to MF59. At this time, few Mo-DCs are loaded with the antigen and it is unlikely that they play a role in the triggering of the T cell response.

Our study is focused on the mode of action of adjuvant MF59, but additional studies are required to understand if the findings described here are also valid for other emulsion adjuvants approved for human vaccination, such as AS03[2–4, 10]. However, consistent with our findings, an AS03 induced increase of antigen-loaded monocytes within the dLN has been described[29], suggesting that a similar process may be promoted by this other emulsion adjuvant.

Beyond what is the role of Mo-DCs in the adjuvanticity of MF59, we believe that our results are very relevant to shed a light on the mode of action of emulsion adjuvants. The data presented in this study contribute to create an emerging vision by which emulsion-adjuvants act through multiple mechanisms and have a multifactorial role in the overall picture of the immune response induced by vaccines containing this type of adjuvants. Emulsions-based adjuvants can play a critical role in improving the efficacy of human vaccines, however, only two squalene-based emulsion adjuvants, MF59 and AS03, are currently approved for human use in influenza vaccines[2–4, 10]. Mechanistic insights into licensed emulsion adjuvants, especially on the role of DCs, can be fundamental to develop new improved emulsion-adjuvanted human vaccines[1, 8, 9, 18, 20]. Thus, our study offers a solid contribution to the challenging objective to produce the most efficacious vaccines possible, with an optimized risk/benefit balance, in order to have a positive impact on human global health through vaccination programs.

## Materials and methods

### Mice

Pathogen-free C57BL/6 (CD45.2), Ly5 (C57BL/6, CD45.1) and OT-II (C57BL/6 background, CD45.2) female mice, aged 6/8 weeks, purchased from Charles River Laboratory, were used.

### Study approval

The animal experiments were approved by local Novartis Animal Welfare Body and performed in compliance with the European directive and Italian law. The code of the approved mouse project are: AEC201111 (starting date November 25th 2011) and AWB2015-01 (starting date August 6th 2015). Mice were sacrificed by cervical dislocation. Surgery for two-photon microscopy studies was conducted as reported in the specific paragraph of Materials and Methods section.

### Antigens, adjuvants and antibodies

#### Antigens

R-Phycoerythrin (PE) (Molecular Probes, Invitrogen-Life Technologies, #P801) was used at 6 μg per dose; OVA (Invitrogen-Life Technologies) was used at 10 μg per dose.

#### Adjuvants

MF59 is Novartis proprietary oil-in-water emulsion consisting of 4.3% (vol/vol) squalene, 0.5% Tween 80, and 0.5% Span 85, in citrate buffer (10 mM). MF59 was prepared by homogenization at 12,000 psi with a Microfluidizer, model 110Y (Microfluidics). The emulsion was sterilized by passage through a polysulfone filter with 0.22 μm pore size (Gelman Sciences) and stored at 4°C. The mean particle size of the emulsion droplets determined with a Mastersizer X (Malvern Instruments) was 194±76 nm. Fluorescently labelled-MF59 was prepared formulating chloroform dissolved DiO fluorescent lipophilic tracer (Molecular Probes; Invitrogen-Life Technologies) in the squalene phase of MF59. The complete evaporation of chloroform was performed before homogenization of the emulsion as described above. The final concentration of DiO was 0.25 μg/ml.

Formulation with MF59 or DiO fluorescently labeled MF59 was prepared diluting the emulsion ½ in the volume dose with physiologic solution. The volume of the immunization dose was 10 μl.

Formulation with ALUM was prepared diluting, ALUM (2 mg/ml) in a solution (prepared with Water For Injection) with 10 mM histidine buffer pH 6.5 and 136 mM NaCl. The volume of the immunization dose was 20 μl.

#### Antibodies

anti-CD11c_APC (BD-Pharmigen; clone HL3); anti-F4/80_eFluor 450 (eBioscence; clone BM8); anti-CD8α_PE-Texas-Red or _R-PE (Invitrogen-Life Technologies; clone 5H10); anti-CD64_Alexa Fluor 488 (R&D Systems; clone 290322); anti-CD80_FITC (BD-Pharmigen; clone 16-10A1); anti-Ly6-C_Alexa Fluor 488 (eBioscience; clone HK1.4); anti-CD86_FITC (BD-Pharmingen; clone GL1); anti-MHC class II I-A/I-E_FITC (BD-Pharmingen; clone 2G9); anti-CD3_PerCp (BD-Pharmigen; clone 145-2C11); anti-CD4_FITC (BD-Pharmingen; clone RM4-5); anti-CD8_Pacific Blue (BD-Pharmigen; clone 53–6.7); anti-CD19_APC (BD Pharmigen; clone 1D3); anti-CD3_BV605 (BD-Pharmingen; clone 145-2C11); anti-CD45.1_APCCy7 (BD-Pharmingen; clone A20); anti-CD45.2_Alexa Fluor 700 (eBioscience; clone 104). Each antibody was titrated to determine the optimal labelling concentration for flow cytometry or confocal microscopy. Relative isotype controls of the same companies were used as negative controls. For flow cytometry, antibodies were firstly titrated individually against the relative isotype control, using LN cells of naïve mice. Then the optimal antibody dilution calculated for each antibody type was used to bind the antibodies to compensation beads (Anti-Rat and Anti-Hamster Ig k/Negative Control Compensation Particle Set, Beckton Dickinson). The same voltage set up for each fluorescence channel used for the antibody titration was applied to acquire compensation beads. Labeled compensation beads were acquired and the compensation was calculated automatically by the software. Then the experiments were run in the same instruments where the compensation was set up, labeling the LN cells with the antibody mix or the corresponding mix of isotype controls. Before to set the gating strategy, all the compensations for each couple of fluorochromes were carefully checked to verify that they were calculated correctly by the software and no over or under compensations were present.

### Immunizations

Flow cytometry and confocal microscopy analysis: mice (3 mice per treatment) were immunized between toes (b.t.) of both legs with a dose of PBS (as negative control), PE or PE adjuvanted with plain or fluorescently labelled MF59 (PE + MF59) and popliteal dLNs were collected 15 minutes, 8 hours, 18 hours and 3 days after the treatment.

Alternatively, mice (3 per treatment) were immunized b.t. in both legs with a dose of PE or PE + MF59 and popliteal dLNs were collected 8 hours after the treatment. LNs from the right legs were immediately processed as described later (flow cytometry analysis indent), whereas each whole LN of the left legs was cultured intact 10 hours in 2 ml (24-well plate) of RPMI-1640 medium (Gibco, Invitrogen-Life Technologies) supplemented with 10% FCS (HyClone) and 1% PSG (EuroClone). After the culture these LNs were processed in the same way (flow cytometry analysis indent). In addition, mice (3 per treatment) were immunized b.t. or intramuscularly (i.m) in the calf muscle (right leg) and the popliteal dLNs were collected 18 hours after the treatment; otherwise mice (3 per treatment) were immunized b.t. (right leg) with PBS, PE, PE + MF59 or PE mixed with ALUM (ALUM-PE) and popliteal dLNs were collected 18 hours after the treatment.

Two-photon microscopy intravital imaging: mice (1 mouse per treatment) were injected b.t. in the right leg with a dose of PE or PE + fluorescently labelled MF59 and the intravital imaging of the popliteal dLN was performed for 15 minutes or 1 hour from the injection, respectively.

Two-photon microscopy 3D-organ imaging: mice (3 mice per treatment) were immunized b.t. in the right leg with a dose of PE or PE + fluorescently labelled MF59 and popliteal dLNs were collected 15 minutes, 8 hours and 18 hours after the treatment.

ELISA: mice (10 mice per treatment) were immunized b.t. or i.m. in the calf muscle (right leg) twice 4 weeks apart with PE and PE + MF59. Sera of mice were collected prior the first immunization (pre-immune), 2 weeks after the first immunization (Post I) and 2 weeks after the second immunization (Post II).

### Flow cytometry analysis

LNs from the right legs were collected at the time points indicated and were immediately processed by enzymatic digestion. LNs of each group of mice were pooled in RPMI-1640 medium (Gibco, Invitrogen-Life Technologies) containing 250 αg/ml DNase I (Roche) and 500 μg/ml Liberase Research Grade (Roche) and incubated for 1h at 37°C agitating by pipetting every 15 min. The obtained cellular suspension was collected by centrifugation at 300Xg for 10 min at room temperature. Then the cells were washed with RPMI-1640 medium (repeating the centrifugation) and were suspended in RPMI-1640 medium supplemented with 10% FCS (HyClone), 1% PSG (EuroClone). The obtained dLN cell suspension was filtered with a 70 μm Cell Strainer (Falcon, Becton Dickinson) and counted with a hemacytometer (Bright-Line). 2 million of LN cells were labelled for 20 min at 4°C in dark conditions, in 50 μl of PBS containing Live/Dead Cell Stain Kit yellow (Molecular Probes; Invitrogen-Life Technologies) used according to the manufacturer’s instructions and titrated fluorescent antibodies (as specified in Fig 1). Then labelled cells were washed with PBS by centrifugation at 300Xg for 10 min at room temperature. Washed cells were suspended in 200 μl of PBS and analyzed by flow cytometry using a LSRII instrument (Becton Dickinson). Roughly 500.000 live gated cells were acquired to make a flow cytometry analysis. Acquisition of the samples were performed using DIVA software (Becton Dickinson), whereas the data analysis were performed using either DIVA or FlowJo software (FlowJo LLC).

### Confocal microscopy

LNs from the left legs were collected individually in dry conditions at the time points indicated, immediately frozen in O.C.T. (Tissue-Tek, Sakura) using liquid nitrogen and stored at -80°C until processing. Cryosections of the LNs were obtained with the cryostat CM1950 (Leica) and stained using fluorescent antibodies. The cryosection (50 μm) were cut along the entire organ in order to analyze all the sections of the organs. The cryosection were fixed using PBS, 3% formaldehyde for 10 min at room temperature, washed twice with PBS and permeabilized with PBS, 3% BSA, 1% saponin (permeabilization buffer) for 30 min at room temperature. Tissue sections were then incubated with titrated fluorescent antibodies (as specified in the Fig 3) diluted in permeabilization buffer for 1h at room temperature in the dark. After washing 3 times with permeabilization buffer and once with PBS, stained tissue sections were sealed using Gold Antifade reagent (Invitrogen-life Technologies) and a coverslip. Images were acquired with Axio Observer LSM700 confocal microscope (Zeiss) at 20°C using a Z stack tool.

### Two-photon microscopy

Intravital imaging of real time translocation of PE and fluorescently labelled MF59 to popliteal dLN was performed as previously described[30, 31].

3D-organ imaging of popliteal dLNs from mice immunized with PE and fluorescently labelled MF59 was performed as previously described[31, 32].

### Measurement of anti-PE antibodies

Serum PE-specific total IgG titers were measured by ELISA. Briefly, maxisorp 96-well plates (Nunc) were coated with a PE solution (2.5 μg/ml) in carbonate buffer (100 μl/well) overnight at 4°C. Plates were then blocked by addition of PBS, 3% polyvinylpyrrolidone (PVP) (SERVA) (200 μl/well), incubated for 2 h at 37°C and then washed once with PBS, 0.05% Tween20 (washing buffer). Serial dilutions (3-fold step) of standard and serum samples in PBS, 0.05% Tween20, 1% BSA were added to the wells and incubated for 2 h at 37°C. Plates were then washed 3 times with washing buffer and incubated for 1 h at 37°C with anti-mouse IgG-alkaline phosphatase (Sigma-Aldrich) solution (100 μl/well). After 3 washes the substrate p-nitrophenylphosphate (Sigma-Aldrich) (100 μl/well) was added for 30 min at room temperature. Absorbance at 405 nm was then measured by a plate spectrophotometer (BioTek–ASHI). Antibody titers were expressed as the reciprocal dilution corresponding to a cut-off at OD_405_  =  0.5.

### Sorting of APCs

Mice (Ly5) (5 mice per treatment) were immunized b.t. in both legs with a dose of PBS (as negative control), OVA or OVA adjuvanted with MF59 (OVA + MF59) and popliteal dLNs were collected 18 hours after the treatment. Popliteal dLN cell suspensions were prepared as previously described (“Flow cytometry analysis” paragraph). LN cells, at a cell concentration of 20 million/ml, were labelled, as previously described, to identify APCs, using Live/Dead Cell Stain Kit aqua (Molecular Probes; Invitrogen-Life Technologies) according to the manufacturer’s instructions and titrated fluorescent antibodies (as specified in Fig 1). Labeled dLN cells were washed as previously described, suspended in PBS at a concentration of 20 million/ml and filtered through a 30 μm cell strainer (Becton Dickinson). APCs from dLN cells were sorted using a FACS Aria II instrument (Becton Dickinson) in 0-32-0 sort precision, applying the gating strategies specified in the text. Sorted APCs were collected in 500 μl of RPMI-1640 medium supplemented with 10% FCS (HyClone), 1% PSG (EuroClone).

### T cell proliferation assay

CD4 T cells were isolated from the spleen of OT-II mice (one mouse per assay) preparing splenocytes by meshing the spleen through a 70 μm Cell Strainer (Falcon; Becton Dickinson) and afterwards by MACS negative selection using the CD4+ T cell isolation Kit (Milteny Biotec) according to the manufacturer’s instruction. T cell purity, routinely around 98%, was determined by flow cytometry labeling the cells as previously described with fluorescent anti-CD4, -CD3, -CD8, -CD19 antibodies. One million of purified OVA-specific CD4 T cells were loaded with fluorescent CFSE (Molecular Probes) suspending them in 1 ml of PBS, 1 μM CFSE, for 10 min in dark condition at room temperature. CFSE loading reaction was stopped adding 1 volume of FCS (HyClone) and the cells were washed with 10 volumes of PBS, centrifuging at 300xg for 10 min at room temperature. Purified APCs (2,5x10^5^ cells) were cultured with CFSE-loaded OVA-specific CD4 T cells (5x10^4^ cells) (APC/T cell ratio: 5/1) in 200 μl (96-well plate, U bottom) of RPMI-1640 supplemented with 10% FCS (Hyclone) and 1% PSG (Gibco; Invitrogen-Life Technolgies). After 3 days of culture, CD4 T cell proliferation was assessed by flow cytometry measuring the halving of CFSE fluorescence. Cell cultures were washed with PBS by centrifugation at 300Xg for 10 min at room temperature, labeled with fluorescent anti-CD3, -CD45.1 and CD45.2 (to discriminate T cells from APCs) and washed as previously described. Then cells were suspended in 200 μl PBS and analyzed by flow cytometry using a LSRII instrument (Becton Dickinson).

### Statistics

Statistical analyses are fully described in each figure and were chosen considering the assumption that MF59 enhances the effect we were measuring, based on published literature or previous data obtained in the study.

## Supporting information

S1 Fig Phenotypic characterization of APCs within the LN. Flow cytometry dot plots representing the gating strategy to define APCs within LN cell suspension of naïve mice are shown. Each reported dot plot derives from the previous one as indicated by the gray arrows and the numbers. Cell suspension of popliteal LNs was obtained and labeled as described in Materials and Methods section. (**a**) Live singlet cells were identified (red gates) firstly by morphological features, excluding cellular debris (1), secondly by Live/Dead staining, excluding dead cells (2) and thirdly again by morphology, excluding doublets (3). (**b**) Among live singlet cells, the APC population was identified as the cell subset single or double positive for CD11c and F4/80 (red gate) (4). Among APCs, macrophages (MΦs, in blue) were discriminated as CD11c^low^_F4/80^+^_CD8α^-^ cells, whereas the rest of APCs (essentially DCs) as CD11c^high^_F4/80^-to+^_CD8α^-to+^ (5).
(TIF)

S2 Fig Phenotype of monocytes versus Mo-DCs reveals that Mo-DCs accumulate within the dLN only 18 hrs after the immunization with MF59. (**a**) Flow cytometry dot plots representing the expression of CD8α and CD64 (upper quadrant) or CD8α and CD11c (lower quadrant) in APCs, 8 hrs after the immunization with PE alone. The few CD8α_F4/80 double positive APCs (red cells) have the phenotype of monocytes, being CD64 positive, CD11c negative and Ly6-C^high^ (S3B Fig). (**b**) Flow cytometry histograms of CD11c expression in CD8α_F4/80 double positive APC subset 18 hours after PE immunization, in presence (red thick line) or absence (red thin line) of MF59. When immunizing without MF59 the CD8α_F4/80 double positive APCs display a monocyte phenotype. CD11c MFI is also reported.
(TIF)

S3 Fig Phenotype of intranodal differentiated Mo-DCs. (**a**) Flow cytometry histograms of CD11c expression by the CD8α_F4/80 double positive APC subset which is CD11c negative/low 8 hrs (grey line) but positive 18 hrs (red line) after immunization with MF59. The CD11c MFI of the CD8α_F4/80 APCs at 8 hrs and 18 hrs from the treatment is also reported. (**b**) The flow cytometry histograms of Ly6-C expression (marker to identify monocytes in vivo) reveal that the CD8α_F4/80 double positive APC subset displays a high expression of Ly6-C both at 8 hrs (grey line) and 18 hrs (red line) after the immunization with MF59. Ly6-C MFI is also reported. Similar results were obtained when immunizing in absence of MF59. (**c**) Flow cytometry histograms of CD64 expression by the CD8α_F4/80 double positive APC subset: left histograms are related to 18 hrs after PE immunization in presence (red line) or absence (grey line) of MF59; right histograms are related to immunization with MF59 18 hrs (red line) or 8 hrs (grey line) after the treatment. CD64 MFI is also reported. (**d**) Flow cytometry histograms of CD11c expression by monocytes and Mo-DCs from dLNs collected 8 hrs from the immunization with MF59 and immediately processed for flow cytometry analysis (grey line) or cultured intact for additional 10 hrs and processed for flow cytometry analysis (red line). CD11c MFI is also reported.
(TIF)

S4 Fig Expression of MCH class II molecules by APCs in MF59 immunized mice. Flow cytometry dot plots reporting the profile of MHC class II expression by MΦs (blue, left panel), Mo-DCs (red, central panel) and DCs (green, right panel) after 18 hrs from the immunization with PE + MF59. Representative results of one experiment out of two are shown.
(TIF)

S5 Fig Mo-DCs accumulation within dLNs is observed also upon intramuscular immunization and occurred three days after immunization with ALUM adjuvant. (**a**) Mice were immunized between toes (b.t.) or intramuscularly (i.m.) with PE alone or adjuvanted with MF59. Cell suspensions of popliteal dLNs were analyzed by flow cytometry 18 hrs after the immunization. Flow cytometry dot plots showing the expression of CD8α and F4/80 from mice immunized with PE + MF59 reveal Mo-DC accumulation (red arrows) both after b.t. and i.m. administration. Representative results of one experiment out of two are shown. (**b**) Mice were immunized between toes with PE, PE + MF59 or PE-ALUM. Cell suspensions of popliteal dLNs were analyzed by flow cytometry 18 hrs after the immunization. Flow cytometry dot plots showing the expression of CD8α and F4/80 reveal that Mo-DCs did not accumulate after the administration of PE adjuvanted with ALUM (PE—ALUM) in contrast with MF59 (red arrow). Representative results of one experiment out of two are shown. (**c**) Similar experiment as described in panel (b), but popliteal dLNs were collected 18 hrs, 3 d and 7 d after the immunization. Flow cytometry dot plots reveal that the CD8α_F4/80 double positive APC population (red cells) accumulates within the dLN and displays the CD64+ Mo-DC phenotype, only 3 d after the immunization, in presence of ALUM (PE–Alum), whereas at 7 d, this cell subset is almost disappeared. Representative results of one experiment out of two are shown. (**d**) Flow cytometry dot plot of Mo-DCs from the experiment reported in panel (c) reveals that 3 days after the immunization with ALUM, Mo-DCs are almost antigen free, because roughly 2% of the cells are loaded with PE. A representative result of one experiment out of two is shown.
(TIF)

S6 Fig MF59 promotes the persistence of antigen-loaded APCs within the dLN during the first 18 hours. (**a**) Flow cytometry dot plots of PE-positive dLN cells (left dot plot) and PE-positive APCs (central and right dot plots) 18 hrs after immunization, identified according to the gating strategy reported in S1 Fig, are shown. The majority of PE-loaded dLN cells (red gate) is formed by APCs (red dotted gates) both in presence (central dot plot) or absence (right dot plot) of MF59, as indicated also by the reported percentage of APCs (% in red). Representative results of one experiment out of three are shown. (**b**) Graph bar histogram report the percentage (upper graph) and the number (lower graph) of PE-positive APCs, in the time-course study depicted in Fig 1, at the indicated time points after immunization with PE (white bars) or PE + MF59 (grey bars). Results from three independent experiments are plotted. Statistical analysis: parametric one-tailed T-test between PE and PE+MF59 conditions per each time point has been applied to calculate the p-value. **P<0.01.
(TIF)

S7 Fig Mo-DCs are mostly double loaded with PE and MF59 compared to DCs and MΦs. Graph bar histogram report the average percentage (+ standard deviation) of MΦs (blue bar), DCs (green bar) and Mo-DCs (red bar) double positive for PE and MF59 (PE/MF59) 18 hours after the immunization. Results of two independent experiments are plotted. Statistical analysis: parametric one-way ANOVA test (Dunnett’s multiple comparison using Mo-DC as control column) has been applied to calculate the P-value. *P<0.05. **P<0.01.
(TIF)

S8 Fig Only Mo-DCs display up-regulation of CD86 co-stimulatory molecule. Flow cytometry histograms of CD86 expression by MΦs (blue), DCs (green) and Mo-DCs (red) from mice immunized with PE (thin lines) or PE + MF59 (thick lines). The MFI of CD86 expression is reported per each APC subset. Representative results of one experiment out of two are reported.
(TIF)

S9 Fig APCs from dLNs of mice immunized with MF59 enhance the triggering of antigen-specific T cell response. (**a**) The bar graph histogram shows the percentage of CFSE halving (which measures the proliferating cells) of OT-II OVA-specific CD4 T cells cultured with APCs derived from dLNs of mice collected 18 hrs after immunization with with OVA (white bar) or OVA + MF59 (grey bar). Data from two independent experiments are plotted. Statistical analysis: parametric one-tailed T-test was used to calculate the P-value. *P˂0.05. (**b**) Flow cytometry histograms of CFSE halving of OT-II CD4 T cells in one of the two experiments plotted in (a).
(TIF)

S10 Fig MF59 works as adjuvant using PE as model antigen. Graph reports the anti-PE IgG titers in the mouse sera collected two weeks after the second immunization with PE (white dots) or PE + MF59 (black dots) in two independent experiments, as indicated (exp. 1; exp. 2). Each dot depicts the antibody titer from a single mouse. The arithmetic mean of the values of the antibody titers of each mouse is indicated by a black horizontal line. Statistical analysis: parametric one-tailed T-test was used to calculate the P-value. *P˂ 0.05.
(TIF)

S1 Video Real time translocation to popliteal dLN of PE injected between toes. Mice were injected b.t. with PE and a real time intravital imaging analysis of the popliteal dLN was taken 15 minutes from the injection (procedure described in Materials and Methods section). The time-lapse of the antigen translocation to popliteal dLN is reported. In red is shown the fluorescence of PE whereas in blue is shown the fluorescence of the LN collagen capsule. The time-frame between each snap-shot is 15 seconds. After b.t. injection the translocation of antigen to popliteal dLN is very rapid (less than a minute). Representative result of one experiment out of two is shown.
(MP4)

S2 Video Real time translocation to popliteal dLN of PE and MF59 injected between toes. Mice were injected b.t. with PE + fluorescently labelled-MF59 and a real time intravital imaging analysis of the popliteal dLN was taken 1 hour from the injection (procedure described in Materials and Methods section). The time-lapse of the antigen translocation to popliteal dLN is reported. Elapsed time is shown as hours:minutes:seconds. In red is shown the fluorescence signal of PE, in green is shown the fluorescence of MF59 whereas in blue is shown the fluorescence of the LN collagen capsule. The time-frame between each snap-shot is 30 seconds. After injection between fingers the translocation of antigen and MF59 to popliteal dLN is very rapid (less than a minute). Representative result of one experiment out of two is shown.
(MP4)

S3 Video 3D antigen localization within the dLN: 18 hrs after injection the antigen is preferentially accumulated within the medullary compartment of the LN. Mice were immunized b.t. with PE and popliteal dLNs were collected, processed and analyzed by two-photon microscopy (all the procedures are described in Materials and Methods section). 3D-imaging of the LN is shown in the video. PE (red) and collagen (blue) signals are reported. The medullary area is distinguished by the presence of the efferent lymphatic vessel (white arrow). Representative result of one experiment out of three is reported.
(MP4)

S4 Video 3D antigen and MF59 localization within the dLN: 18 hrs after injection the antigen is preferentially, whereas MF59 is totally, accumulated within the medullary compartment of the LN. Mice were immunized b.t. with PE and fluorescently labelled-MF59 and popliteal dLNs were collected, processed and analyzed by two-photon microscopy (all the procedures described in Materials and Methods section). 3D-imaging of the LN is shown in the video. PE (red), MF59 (green) and collagen (blue) signals are reported. The medullary area is distinguished by the presence of the efferent lymphatic vessel (white arrow). Representative result of one experiment out of three is reported.
(MP4)
