# Supplementary figures and images for: Vaccine adjuvant MF59 promotes the intranodal differentiation of antigen-loaded and activated monocyte-derived dendritic cells
Source: PLoS One. 2017 Oct 31;12(10):e0185843. doi: 10.1371/journal.pone.0185843 (PMC5663329; doi:10.1371/journal.pone.0185843)

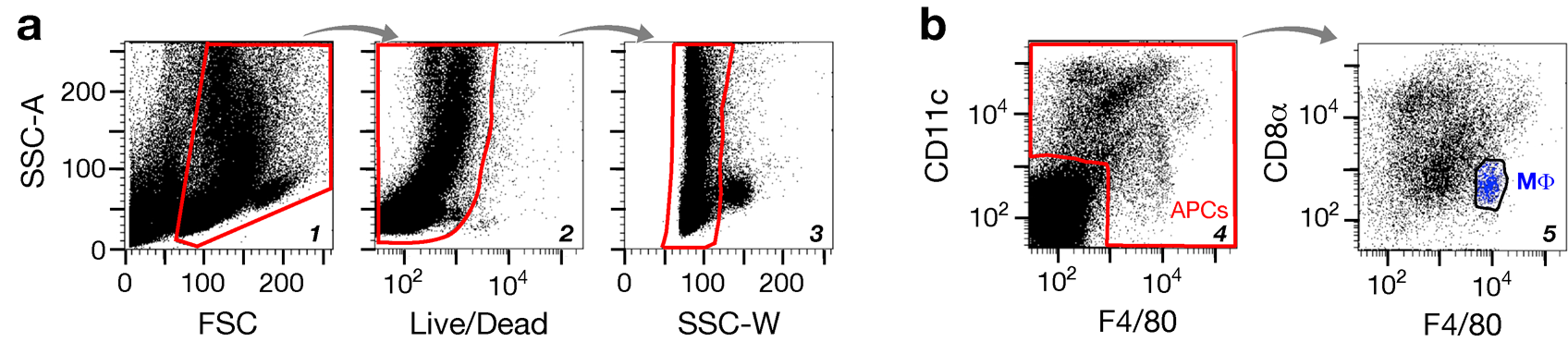

Supplement: S1 Fig — Flow cytometry dot plots representing the gating strategy to define APCs within LN cell suspension of naïve mice are shown. Each reported dot plot derives from the previous one as indicated by the gray arrows and the numbers. Cell suspension of popliteal LNs was obtained and labeled as described in Materials and Methods section. (a) Live singlet cells were identified (red gates) firstly by morphological features, excluding cellular debris (1), secondly by Live/Dead staining, excluding dead cells (2) and thirdly again by morphology, excluding doublets (3). (b) Among live singlet cells, the APC population was identified as the cell subset single or double positive for CD11c and F4/80 (red gate) (4). Among APCs, macrophages (MΦs, in blue) were discriminated as CD11clow_F4/80+_CD8α- cells, whereas the rest of APCs (essentially DCs) as CD11chigh_F4/80-to+_CD8α-to+ (5). (TIF) [file pone.0185843.s001.tif]

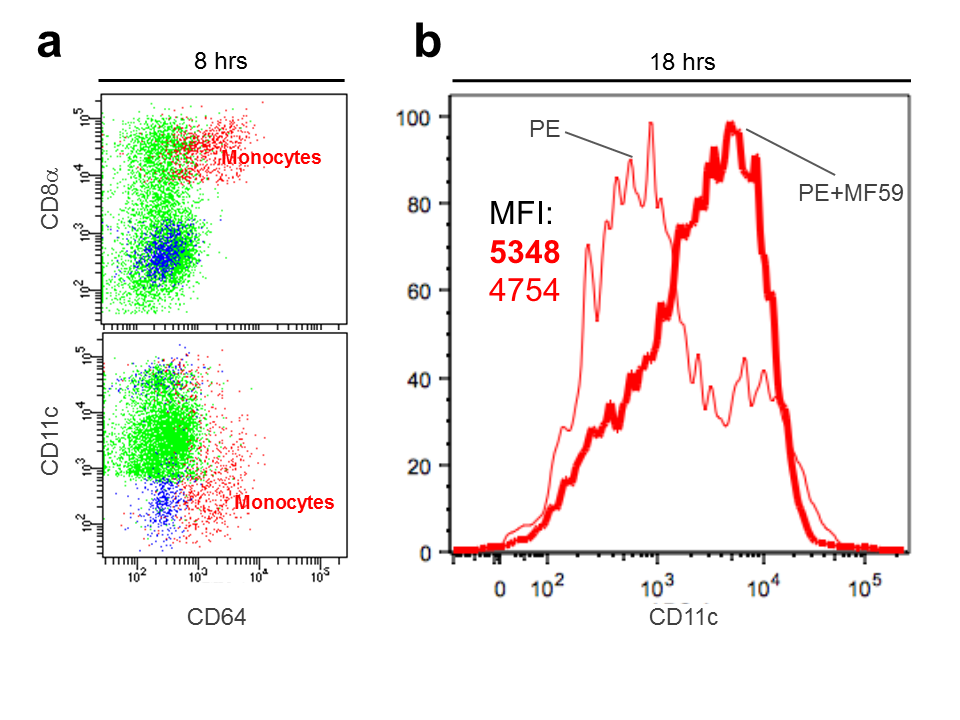

Supplement: S2 Fig — (a) Flow cytometry dot plots representing the expression of CD8α and CD64 (upper quadrant) or CD8α and CD11c (lower quadrant) in APCs, 8 hrs after the immunization with PE alone. The few CD8α_F4/80 double positive APCs (red cells) have the phenotype of monocytes, being CD64 positive, CD11c negative and Ly6-Chigh (S3B Fig). (b) Flow cytometry histograms of CD11c expression in CD8α_F4/80 double positive APC subset 18 hours after PE immunization, in presence (red thick line) or absence (red thin line) of MF59. When immunizing without MF59 the CD8α_F4/80 double positive APCs display a monocyte phenotype. CD11c MFI is also reported. (TIF) [file pone.0185843.s002.tif]

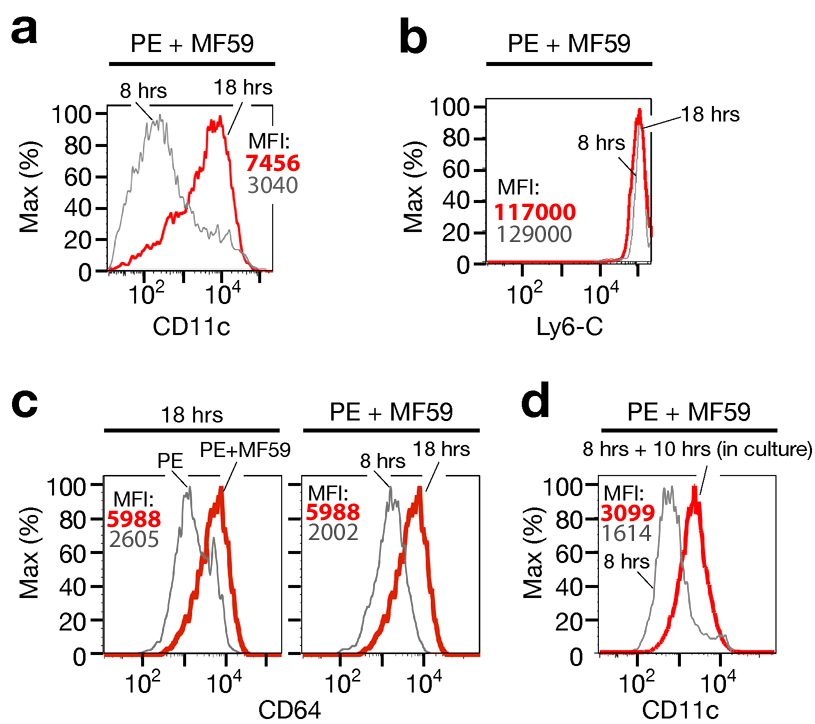

Supplement: S3 Fig — (a) Flow cytometry histograms of CD11c expression by the CD8α_F4/80 double positive APC subset which is CD11c negative/low 8 hrs (grey line) but positive 18 hrs (red line) after immunization with MF59. The CD11c MFI of the CD8α_F4/80 APCs at 8 hrs and 18 hrs from the treatment is also reported. (b) The flow cytometry histograms of Ly6-C expression (marker to identify monocytes in vivo) reveal that the CD8α_F4/80 double positive APC subset displays a high expression of Ly6-C both at 8 hrs (grey line) and 18 hrs (red line) after the immunization with MF59. Ly6-C MFI is also reported. Similar results were obtained when immunizing in absence of MF59. (c) Flow cytometry histograms of CD64 expression by the CD8α_F4/80 double positive APC subset: left histograms are related to 18 hrs after PE immunization in presence (red line) or absence (grey line) of MF59; right histograms are related to immunization with MF59 18 hrs (red line) or 8 hrs (grey line) after the treatment. CD64 MFI is also reported. (d) Flow cytometry histograms of CD11c expression by monocytes and Mo-DCs from dLNs collected 8 hrs from the immunization with MF59 and immediately processed for flow cytometry analysis (grey line) or cultured intact for additional 10 hrs and processed for flow cytometry analysis (red line). CD11c MFI is also reported. (TIF) [file pone.0185843.s003.tif]

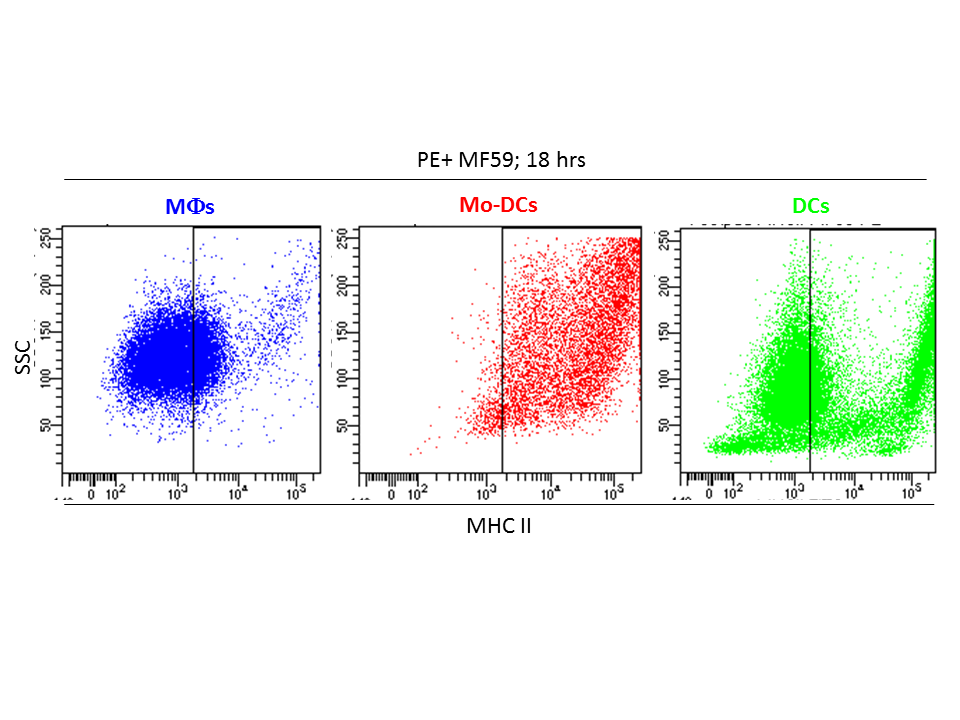

Supplement: S4 Fig — Flow cytometry dot plots reporting the profile of MHC class II expression by MΦs (blue, left panel), Mo-DCs (red, central panel) and DCs (green, right panel) after 18 hrs from the immunization with PE + MF59. Representative results of one experiment out of two are shown. (TIF) [file pone.0185843.s004.tif]

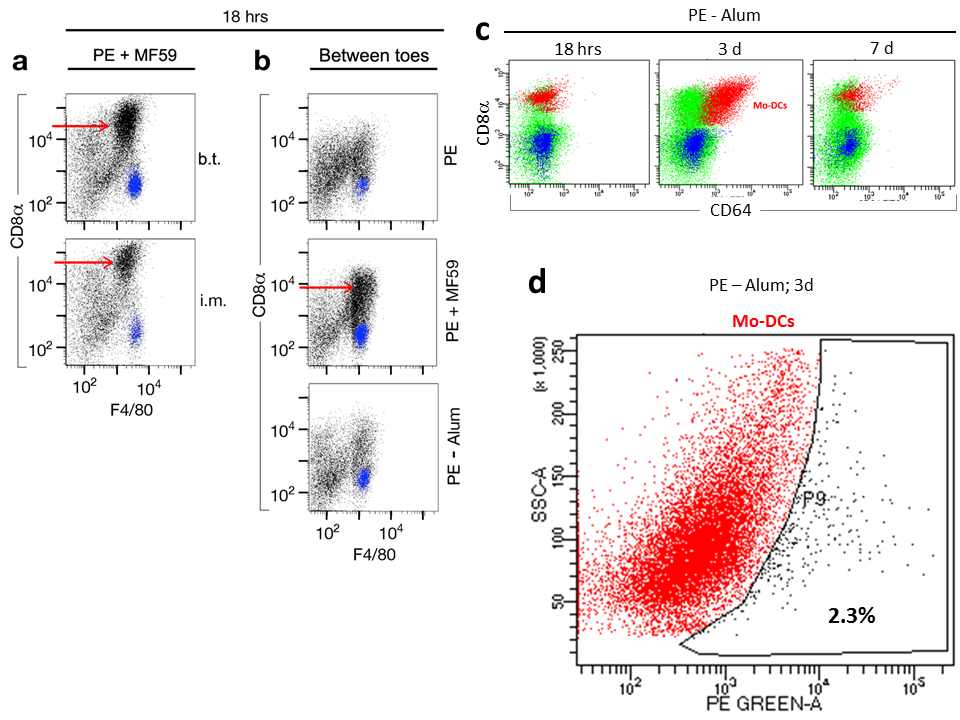

Supplement: S5 Fig — (a) Mice were immunized between toes (b.t.) or intramuscularly (i.m.) with PE alone or adjuvanted with MF59. Cell suspensions of popliteal dLNs were analyzed by flow cytometry 18 hrs after the immunization. Flow cytometry dot plots showing the expression of CD8α and F4/80 from mice immunized with PE + MF59 reveal Mo-DC accumulation (red arrows) both after b.t. and i.m. administration. Representative results of one experiment out of two are shown. (b) Mice were immunized between toes with PE, PE + MF59 or PE-ALUM. Cell suspensions of popliteal dLNs were analyzed by flow cytometry 18 hrs after the immunization. Flow cytometry dot plots showing the expression of CD8α and F4/80 reveal that Mo-DCs did not accumulate after the administration of PE adjuvanted with ALUM (PE—ALUM) in contrast with MF59 (red arrow). Representative results of one experiment out of two are shown. (c) Similar experiment as described in panel (b), but popliteal dLNs were collected 18 hrs, 3 d and 7 d after the immunization. Flow cytometry dot plots reveal that the CD8α_F4/80 double positive APC population (red cells) accumulates within the dLN and displays the CD64+ Mo-DC phenotype, only 3 d after the immunization, in presence of ALUM (PE–Alum), whereas at 7 d, this cell subset is almost disappeared. Representative results of one experiment out of two are shown. (d) Flow cytometry dot plot of Mo-DCs from the experiment reported in panel (c) reveals that 3 days after the immunization with ALUM, Mo-DCs are almost antigen free, because roughly 2% of the cells are loaded with PE. A representative result of one experiment out of two is shown. (TIF) [file pone.0185843.s005.tif]

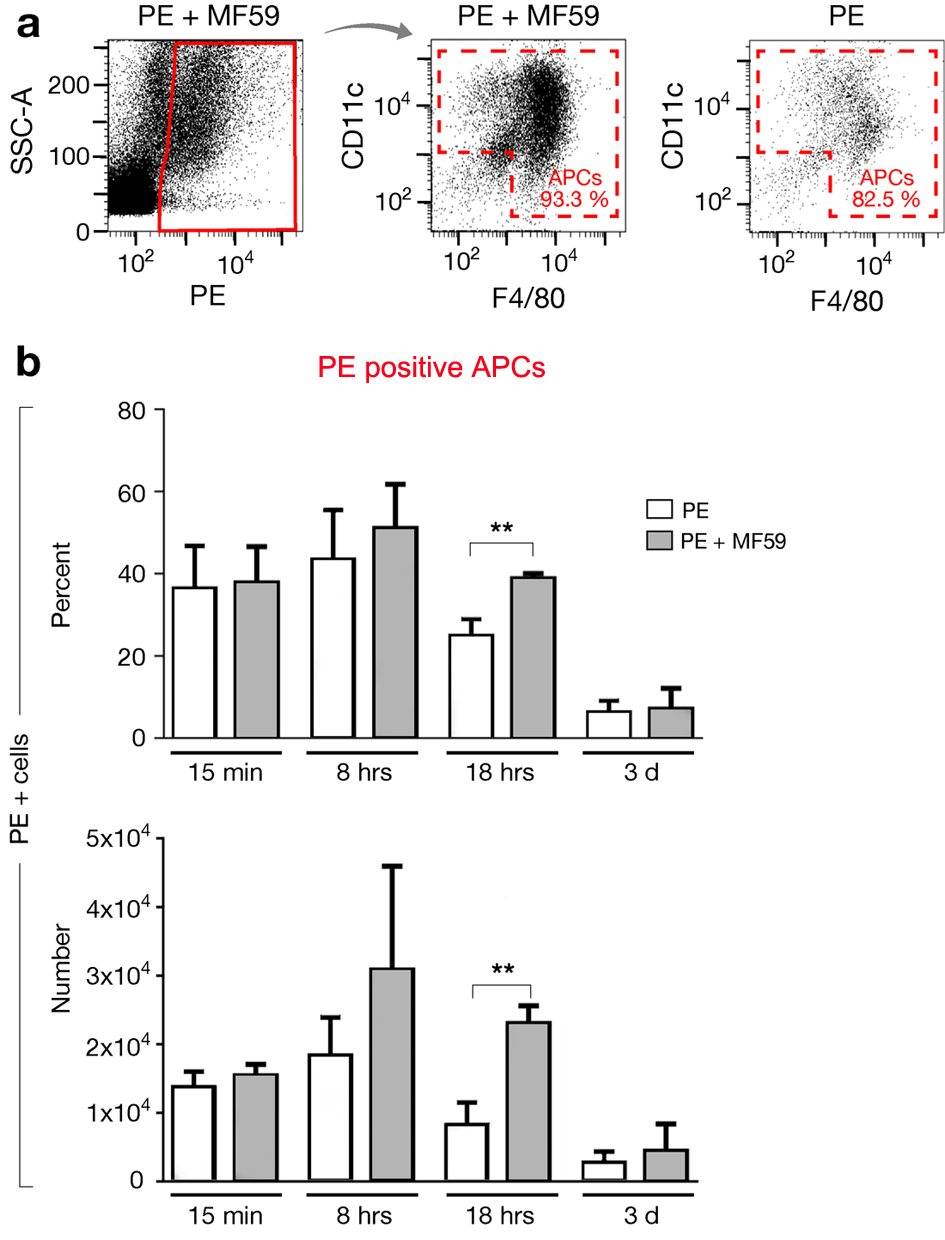

Supplement: S6 Fig — (a) Flow cytometry dot plots of PE-positive dLN cells (left dot plot) and PE-positive APCs (central and right dot plots) 18 hrs after immunization, identified according to the gating strategy reported in S1 Fig, are shown. The majority of PE-loaded dLN cells (red gate) is formed by APCs (red dotted gates) both in presence (central dot plot) or absence (right dot plot) of MF59, as indicated also by the reported percentage of APCs (% in red). Representative results of one experiment out of three are shown. (b) Graph bar histogram report the percentage (upper graph) and the number (lower graph) of PE-positive APCs, in the time-course study depicted in Fig 1, at the indicated time points after immunization with PE (white bars) or PE + MF59 (grey bars). Results from three independent experiments are plotted. Statistical analysis: parametric one-tailed T-test between PE and PE+MF59 conditions per each time point has been applied to calculate the p-value. **P<0.01. (TIF) [file pone.0185843.s006.tif]

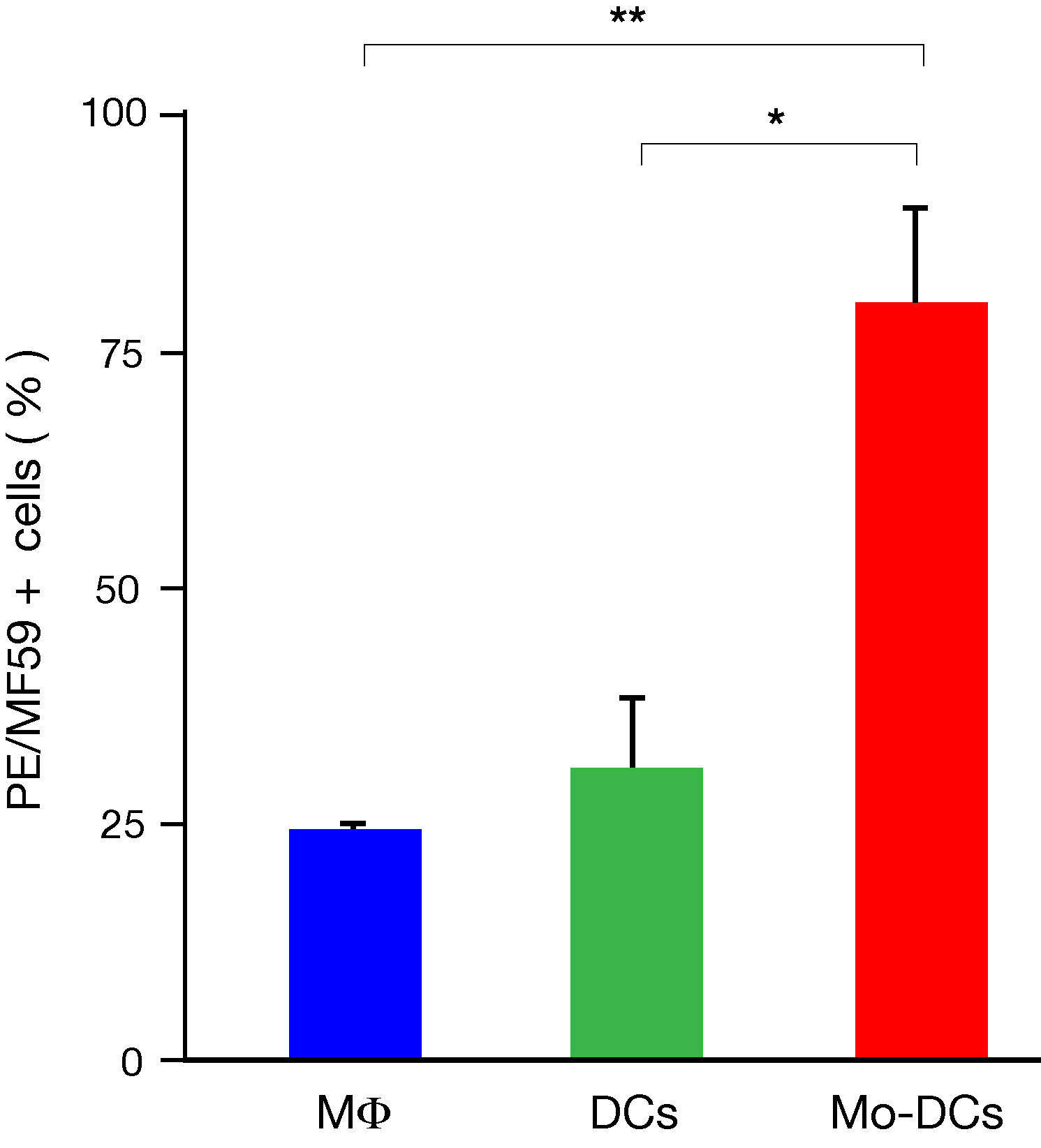

Supplement: S7 Fig — Graph bar histogram report the average percentage (+ standard deviation) of MΦs (blue bar), DCs (green bar) and Mo-DCs (red bar) double positive for PE and MF59 (PE/MF59) 18 hours after the immunization. Results of two independent experiments are plotted. Statistical analysis: parametric one-way ANOVA test (Dunnett’s multiple comparison using Mo-DC as control column) has been applied to calculate the P-value. *P<0.05. **P<0.01. (TIF) [file pone.0185843.s007.tif]

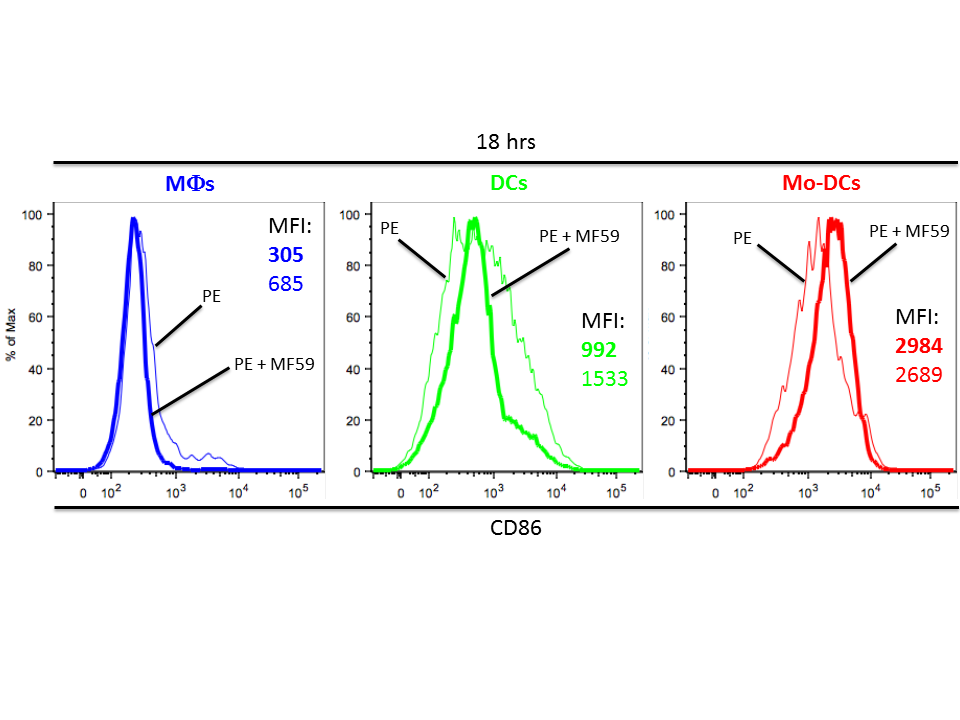

Supplement: S8 Fig — Flow cytometry histograms of CD86 expression by MΦs (blue), DCs (green) and Mo-DCs (red) from mice immunized with PE (thin lines) or PE + MF59 (thick lines). The MFI of CD86 expression is reported per each APC subset. Representative results of one experiment out of two are reported. (TIF) [file pone.0185843.s008.tif]

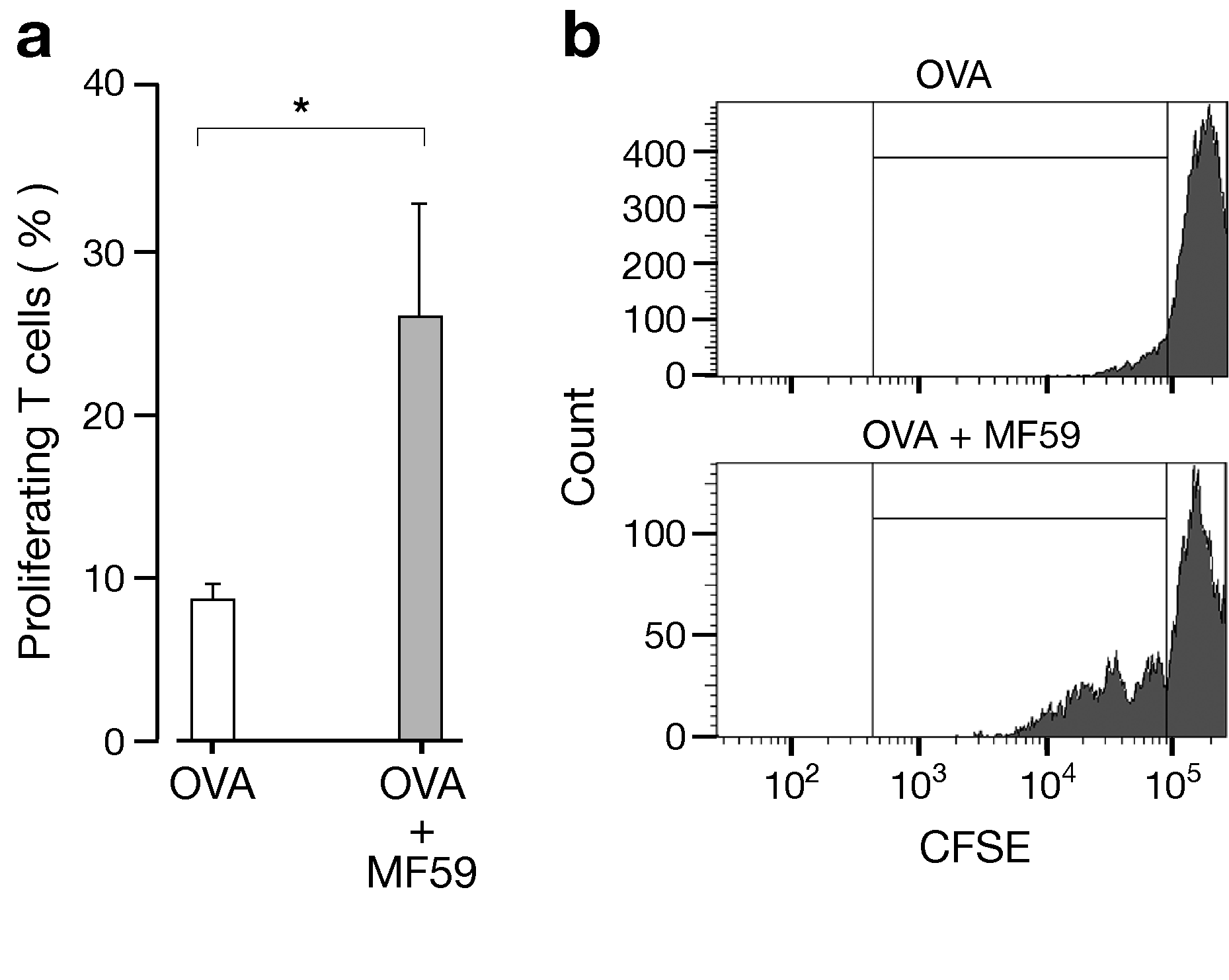

Supplement: S9 Fig — (a) The bar graph histogram shows the percentage of CFSE halving (which measures the proliferating cells) of OT-II OVA-specific CD4 T cells cultured with APCs derived from dLNs of mice collected 18 hrs after immunization with with OVA (white bar) or OVA + MF59 (grey bar). Data from two independent experiments are plotted. Statistical analysis: parametric one-tailed T-test was used to calculate the P-value. *P˂0.05. (b) Flow cytometry histograms of CFSE halving of OT-II CD4 T cells in one of the two experiments plotted in (a). (TIF) [file pone.0185843.s009.tif]

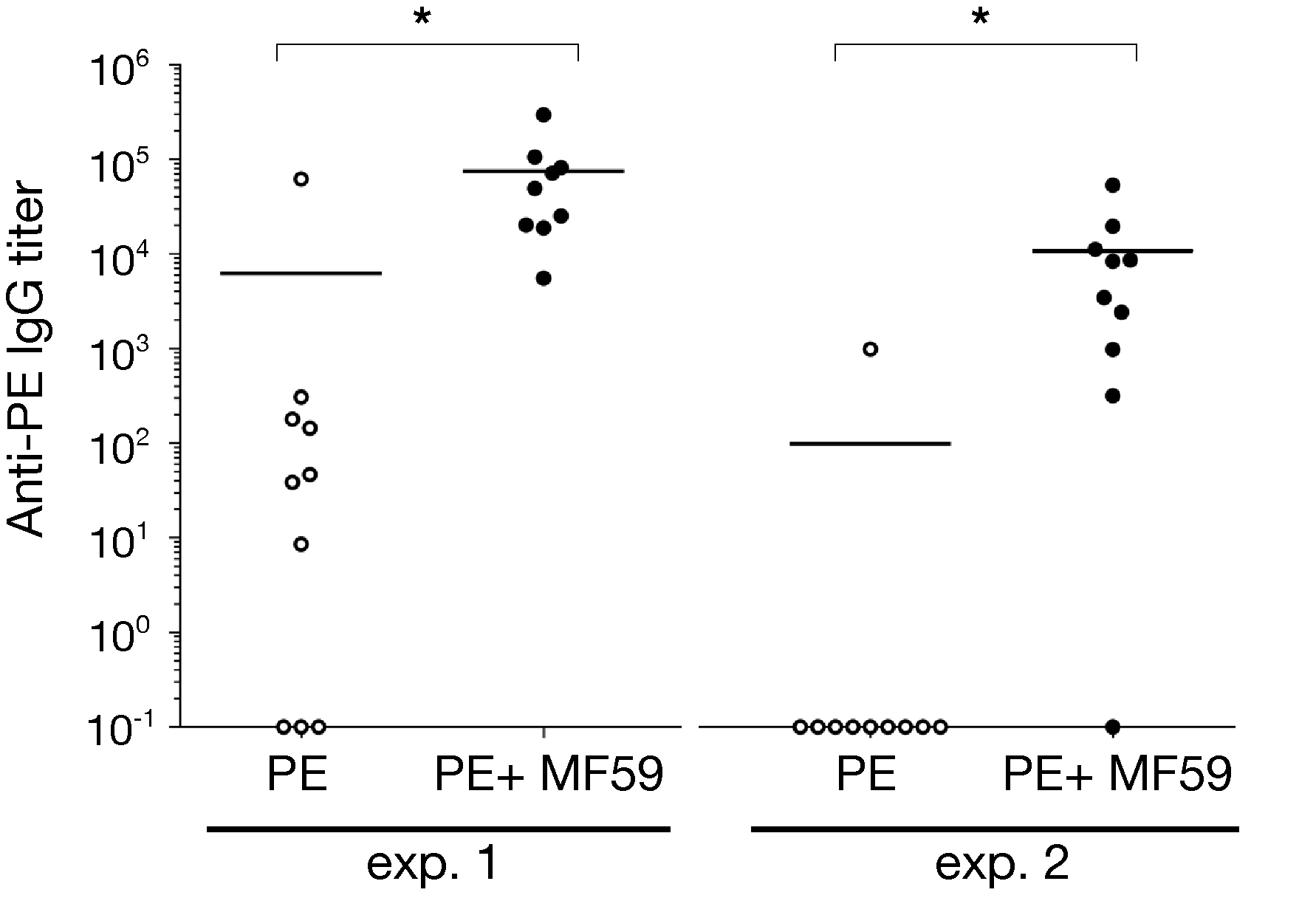

Supplement: S10 Fig — Graph reports the anti-PE IgG titers in the mouse sera collected two weeks after the second immunization with PE (white dots) or PE + MF59 (black dots) in two independent experiments, as indicated (exp. 1; exp. 2). Each dot depicts the antibody titer from a single mouse. The arithmetic mean of the values of the antibody titers of each mouse is indicated by a black horizontal line. Statistical analysis: parametric one-tailed T-test was used to calculate the P-value. *P˂ 0.05. (TIF) [file pone.0185843.s010.tif]
